# Seasonal and Spatial Dynamics of the Primary Vector of *Plasmodium knowlesi* within a Major Transmission Focus in Sabah, Malaysia

**DOI:** 10.1371/journal.pntd.0004135

**Published:** 2015-10-08

**Authors:** Meng L. Wong, Tock H. Chua, Cherng S. Leong, Loke T. Khaw, Kimberly Fornace, Wan-Yusoff Wan-Sulaiman, Timothy William, Chris Drakeley, Heather M. Ferguson, Indra Vythilingam

**Affiliations:** 1 Parasitology Department, Faculty of Medicine, University of Malaya, Kuala Lumpur, Malaysia; 2 Department of Pathobiology and Medical Diagnostics, Faculty of Medicine and Health Sciences, Universiti Sabah Malaysia, Kota Kinabalu, Sabah, Malaysia; 3 Faculty of Infectious and Tropical Diseases, London School of Hygiene and Tropical Medicine, London, United Kingdom; 4 Jesselton Medical Centre, Kota Kinabalu, Sabah, Malaysia; 5 Institute of Biodiversity, Animal Health and Comparative Medicine, University of Glasgow, Scotland, United Kingdom; Liverpool School of Tropical Medicine, UNITED KINGDOM

## Abstract

**Background:**

The simian malaria parasite *Plasmodium knowlesi* is emerging as a public health problem in Southeast Asia, particularly in Malaysian Borneo where it now accounts for the greatest burden of malaria cases and deaths. Control is hindered by limited understanding of the ecology of potential vector species.

**Methodology/Principal Findings:**

We conducted a one year longitudinal study of *P*. *knowlesi* vectors in three sites within an endemic area of Sabah, Malaysia. All mosquitoes were captured using human landing catch. *Anopheles* mosquitoes were dissected to determine, oocyst, sporozoites and parous rate. *Anopheles balabacensis* is confirmed as the primary vector of. *P*. *knowlesi* (using nested PCR) in Sabah for the first time. Vector densities were significantly higher and more seasonally variable in the village than forest or small scale farming site. However *An*. *balabacensis* survival and *P*. *knowlesi* infection rates were highest in forest and small scale farm sites. *Anopheles balabacensis* mostly bites humans outdoors in the early evening between 1800 to 2000hrs.

**Conclusions/Significance:**

This study indicates transmission is unlikely to be prevented by bednets. This combined with its high vectorial capacity poses a threat to malaria elimination programmes within the region.

## Author Summary

The first natural infection of *Plasmodium knowlesi* was reported 40 years ago. At that time it was perceived that the infection would not affect humans. However, now *P*. *knowlesi* is the predominant malaria species (38% of the cases) infecting people in Malaysia and is a notable obstacle to malaria elimination in the country. *Plasmodium knowlesi* has also been reported from all countries in Southeast Asia with the exception of Lao PDR and Timor Leste. In Sabah, Malaysian Borneo cases of human *P*. *knowlesi* are increasing. Thus, a comprehensive understanding of the bionomics of the vectors is required so as to enable proper control strategies. Here, we conducted a longitudinal study in Kudat district, Sabah, to determine and characterize the vectors of *P*. *knowlesi* within this transmission foci. *Anopheles balabacensis* was the predominant mosquito in all study sites and is confirmed as vector for *P*. *knowlesi* and other simian malaria parasites. The peak biting time was in the early part of the evening between1800 to 2000. Thus, breaking the chain of transmission is an extremely challenging task for the malaria elimination programme.

## Introduction

Significant progress has been made fighting malaria in the last decade, decreasing the incidence of cases and mortality by 30% and 47% respectively on a global scale [1] and reducing cases by 76% in Asia Pacific countries [2]. The development and use of better tools for diagnostics and treatment [3] coupled with substantial increases in the coverage of vector control methods such as Long Lasting Insecticide Treated bednets (LLINs) and Indoor Residual Spraying [4] have contributed to these successes.

An additional challenge to malaria elimination is the existence of a zoonotic reservoir of malaria. The primate malaria *Plasmodium knowlesi* has recently been documented as causing human infections in multiple countries in Southeast Asia [5–11], and is a serious public health problem within Malaysia [12–19]. In the Malaysian state of Sabah, this parasite is now responsible for the greatest number of malaria cases with 815 and 996 cases reported respectively in 2012 and 2013 [20].

This growing burden of *P*. *knowlesi* presents a notable obstacle to malaria elimination in Malaysia where historically, most transmission has been due to human-specific parasite species [17]. Since 2011, Malaysia has made great progress towards the elimination of these human malaria species, leading to a target for complete elimination by 2020 [21]. Whether existing elimination targets can be met in the face of increasing *P*. *knowlesi* cases with this *Plasmodium* now causing 38% of human malaria cases in Malaysia in 2012 remains to be seen.

Two features of *P*. *knowlesi* make it particularly difficult to control by conventional methods: (1) it has a sizeable zoonotic reservoir in macaques, which means that even if infections are eliminated from humans there remains a risk of future spillover, and (2) current evidence indicates that previously incriminated mosquito vectors of *P*. *knowlesi* in Malaysia bite and rest outdoors where control methods such as LLINs and IRS will not be effective [19, 22, 23].

Incrimination of vector species responsible of *P*. *knowlesi* transmission is a crucial first step for planning control but limited data is available on vectors of simian malaria in this region. Mosquitoes belonging to the Anopheles leucosphyrus group are thought to be responsible for *P*. *knowlesi* transmission. *Anopheles hackeri* was the first species to be incriminated as a vector, in the coastal area of Selangor [24], followed by *An*. *latens* in Kapit, Sarawak [22, 25], *An*. *cracens* in Kuala Lipis [14, 23] and *An*. *introlatus* in Hulu Selangor [19]. In Vietnam *An*. *dirus*, was incriminated as the *P*. *knowlesi* vector [26, 27]. The considerable spatial variation in *P*. *knowlesi* vector species both within and beyond Malaysia reinforces the need for detailed studies of vector ecology in a localized context to guide appropriate control strategy.

*Anopheles balabacensis* is hypothesized to be the primary vector of *P*. *knowlesi* within the current, extensive transmission foci of *P*. *knowlesi* in Sabah. Based on extensive studies carried out in the region in the 1980s [28–31] *An*. *balabacensis* was incriminated as a vector of human malaria, and laboratory studies that showed that *An*. *balabacensis* can be experimentally infected with *P*. *knowlesi* [32]. Since this early work, there has been significant ecological change occurring throughout Sabah due to conversion of forest to palm oil plantations [33–35]. How these changes have impacted the abundance, diversity and transmission potential of *P*. *knowlesi* vectors needs to be investigated. Control of *P*. *knowlesi* in Sabah requires confirmation that *An*. *balabacensis* remains the most likely vector, and characterization of its dynamics within a range of habitats that reflect current land use patterns. For that purpose, we conducted a 12 month longitudinal study within the large, ongoing focus of *P*. *knowlesi* transmission in Kudat and Banggi Island (Kudat District) in Sabah, aiming to characterize the abundance and biting behavior of potential vector species and incriminate vector species. These findings will be of importance to guide the development of local vector control programmes to eliminate malaria transmission.

## Methods

### Description of study area

**Fig 1:**
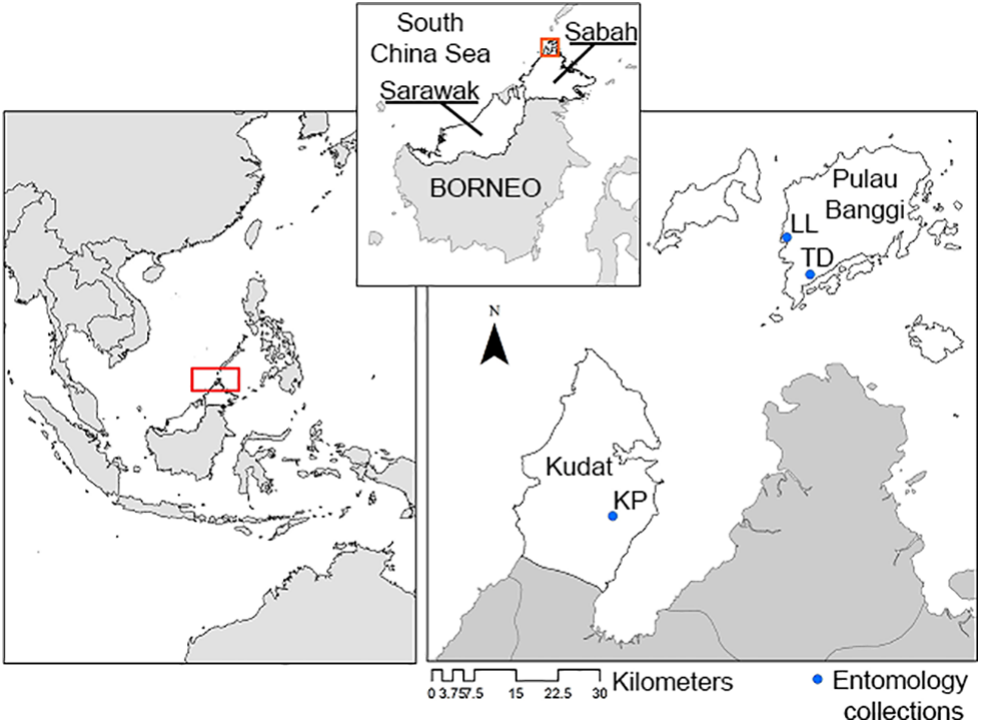
Map showing study sites in Kudat and Banggi Island.

Studies were conducted in three sites: Timbang Dayang (TD) (117°102’92”E, 7°155’85”N) and Limbuak Laut (LL) 117°065’75”E, 7°215’84”N) on Banggi island, and Kampung Paradason (KP) (116°786’35”E, 6°768’37”N on mainland Kudat (Fig 1). These sites were selected to reflect the range of ecotypes broadly representative of the study area in Northern Sabah: small scale farming (TD), secondary forest (LL) and a village settlement (KP). Sightings of macaques and recent human cases of *P*. *knowlesi* were reported near all sites. Timbang Dayang is a village with a population of 180 people. It is situated in a hilly landscape where houses are surrounded by small farming areas ~200 meters from the edge of secondary forest. These small farms (>1 hectare) contain mixed agriculture primarily for household consumption, including maize, banana and fruit trees. The mosquito collection site was near the edge of farm approximately 150 meters from the group of houses.

Limbuak Laut (LL) is a village consisting of 144 people, with houses situated on a road bordering closed canopy secondary forest. Mosquito sampling was conducted at a point situated within the secondary forest, at a distance of approximately 500 meters from the edge of the forest.

Paradason in Kudat is a village of 160 people situated in a heavily cultivated area, characterized by swidden farming and small plantations of rubber and palm oil. The area is undergoing a high rate of environmental change, including frequent burning and clearing of land. Little intact secondary forest remains in this area. The local community lives in both individual houses and a traditional communal longhouse shared between six households. Here mosquitoes were sampled at a point near the longhouse (100m), and in an associated garden area 75m away from the first collecting point.

### Mosquito collection

Mosquitoes were collected by human landing collections (HLC) which were carried out monthly at all sampling sites from August 2013 to July 2014 (three nights per month at TD and LL and two nights in KP). Two men per team carried out collections at each site from 1800 to 0600 hrs. Mosquitoes landing on the legs of catchers were captured individually in vials which were then plugged with cotton wool and labelled by hour and collection sites. A supervisor visited the team hourly to ensure collections were being carried out. In TD and LL, collections were conducted by one team each night, whereas two teams (situated ~75 m apart) worked each night in Kudat. Thus a total of six individual human landing catches were performed each month in TD and LL, and eight per month at KP.

### Mosquito identification and dissection

In the laboratory *Anopheles* mosquitoes were identified using the keys of Reid (1968) and Sallum (2005). Specimens morphologically identified as *An*. *balabacensis* were further confirmed by PCR and sequencing analysis of ITS2 and CO1 genes [19]. *Anopheles* mosquitoes were dissected to extract their ovaries, midguts and salivary glands to determine parity, oocyst and sporozoites respectively. All positive midguts and salivary glands, and the corresponding head and thorax of these positive specimens were transferred into individual microcentrifuge tubes containing 95% ethanol for subsequent molecular analysis.

### DNA extraction and nested PCR assay

Ethanol was allowed to evaporate completely from specimen tubes by placing them in a Thermomixer (Eppendorf, Germany) at 70°C. Genomic DNA was extracted from the guts and glands using the DNeasy tissue kit (Qiagen, Germany) according to the manufacturer’s protocol. The eluted DNA was kept at -20°C until required. A nested PCR was performed to detect and identify human specific malaria parasites (*Plasmodium falciparum*, *P*. *vivax*, *P*. *malariae* and *P*. *ovale*) and *P*. *knowlesi* found in mosquitoes using primers based on the *Plasmodium* small subunit ribosomal RNA (ssurRNA) [12, 36]. Primers and protocol used for human malaria and *P*. *knowlesi* detection were as developed by Singh et al [12] and Lee et al [37] for other simian malaria. Positive and negative controls were also included for each batch of assays.

### Statistical analysis

Statistical analysis was conducted using PASW Statistics 18 and R programming language for statistical analysis (version 3.2.0). Generalised linear mixed models (GLMM) were constructed to analyse the following parameters of interest: the abundance of *An*. *balabacensis*, their time of biting, and the proportion of vectors that were (i) infected with oocysts, (ii) infected with sporozoites, and (iii) that were parous. In all analyses, locality (TD, LL or KP) was fit as a fixed effect. Month was fit alternatively as a fixed (to predict monthly values) or random effect (to test for differences between localities while controlling for seasonal variation).

Poisson and negative binomial distributions were used separately in the analysis of mosquito abundance, while a binomial distribution was assumed in all analysis of proportion data (parity and infection rates). Zero inflation in count data (mosquito abundance) was assessed. Models testing associations between response variables (vector abundance, parity and infection rates) explanatory variables (locality and month) and random effects of sampling night were assessed through comparison on the basis of having higher log-likelihood and lower *Akaike information criterion* (AIC) values, as well as the result of analysis of variance (ANOVA) of nested models). Tukey post hoc contrasts were used to differentiate the nature of statistical differences between localities. Graphs were produced using GraphPad Prism 6.0.

### Ethical clearance

This project was approved by the NMRR Ministry of Health Malaysia (NMRR-12-786-13048). All volunteers who carried out mosquito collections signed informed consent forms and were provided with antimalarial prophylaxis during participation.

## Results

### Species composition of *Anopheles* in different study sites

A total of 1884 *Anopheles* belonging to ten different species was obtained of which *An*. *balabacensis* predominated (95.1% of total, Table 1) in all sites. Other species of *Anopheles* were found in very low numbers and present in one or two localities only. *Anopheles balabacensis* was the only species from the Leucosphyrus group caught. A total of 379 Culicines were obtained but were not identified to species.

**Table 1:** *Anopheles* species collected in study sites in Kudat Division, Sabah from August 2013 to July 2014.

Mosquito species	Banggi Island		Kudat	Total (%)
	Limbuak Laut	Timbang Dayang	Kg Paradason	
*An*. *balabacensis*	479	464	848	1791 (95.1)
*An*. *donaldi*	10	3	12	25 (1.3)
*An*. *vagus*	1	18	0	19 (1.0)
*An*. *umbrosus* group	7	0	0	7 (0.4)
*An*. *barbirostris* group	2	0	11	13 (0.7)
*An*. *tessellatus*	0	0	2	2 (0.1)
*An*. *watsonii*	1	0	2	3 (0.2)
*An*. *flavirostris*	0	5	0	5 (0.3)
*An*. *maculatus*	0	0	8	8 (0.4)
*An*. *aconitus*	0	11	0	11 (0.6)
**Total**	500	501	883	1884 (100)

### *Anopheles balabacensis* abundance over time

**Fig 2:**
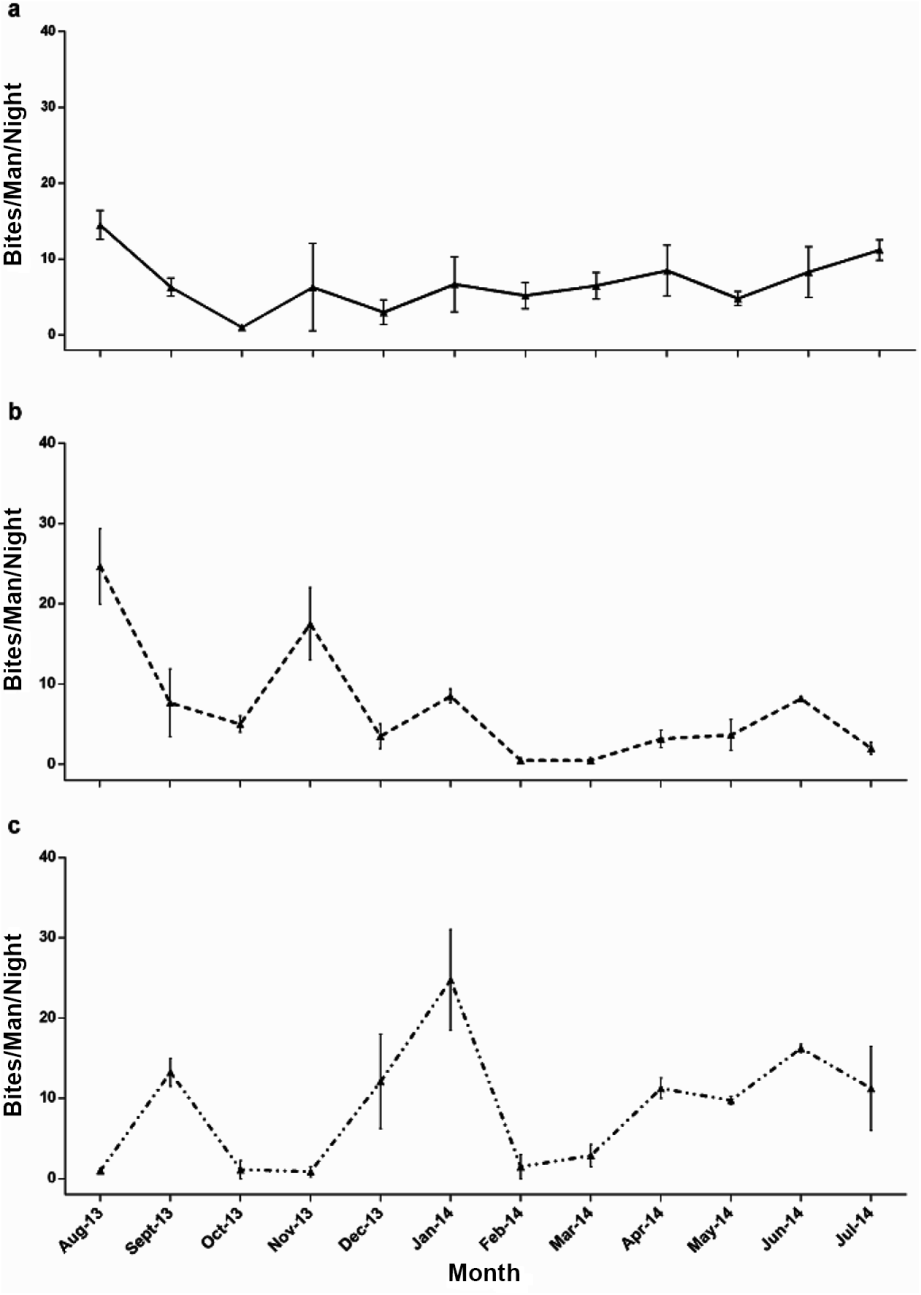
Bites/Man/Night of *An*. *balabacensis* measured each month in three study sites over time. a. Limbuak Laut, b. Timbang Dyang, c. Kg Paradason. Error bars are 95%CI.

The number of *An*. *balabacensis* collected in HLC ranged from ~2–28 per man night, but did not show any clear, consistent trend in seasonality (Fig 2). The pattern of seasonal fluctuation differed between sites (Fig 2). In the forest site (Fig 2A), *An*. *balabacensis* abundance was relatively low (<15 per night) and constant across months. In the small farming site, *An*. *balabaensis* varied more than 10-fold over the course of a year, with a high in August and November, and low from February-to May and July 2014. *Anopheles balabacensis* abundance was more variable in the village settlement (Fig 2C). Here the highest monthly density was observed in January (27 per night) with values <1 per night in October and November.

Analysis using GLMM models indicated that the Poisson distribution was generally a better representation of *An*. *balabacensis* abundance data than the negative binomial. On the basis of statistical models assuming a Poisson distribution, the Tukey post hoc test indicated that *An*. *balabancensis* abundance was significantly higher in the village settlement (KP) than in the two other localities, (KP and LL: (p = 0.04; KP and TD: p = 0.02;Table 2). Controlling for variation across months, *An*. *balabancensis* abundance in the village site was estimated to be ~15–20% higher than in the other localities.

**Table 2:** Generalised linear mixed model fitting of the data. The model used is of the form “glmm<-glmmadmb (parameter ~ locality+(1|month), zero Inflation = T, data = data,family = "pdf")”. KP = Kampung Paradason, LL = Limbuak Laut, TD = Timbang Dayang. AIC *=* Akaike information criterion. Means with different superscript letters indicate they are significantly different.

Parameter	n	Zero-inflatio n	Log-lik-hood	AIC	Mean predicted values			Tukey’s test between means
					KP	LL	TD	
**Bites per man/night**	92	False	-357.36	722.72	7.84^a^	6.26^b^	6.13 ^b^	KP-LL; p = 0.04KP-TD; p = 0.02
**Sporozoite rate**	83	True	-57.93	125.86	0.01 ^a^	0.04 ^b^	0.02 ^ab^	KP-LL; p = 0.04
**Oocyst rate**	83	True	-59.298	128.59	0.01 ^a^	0.03 ^ab^	0.03 ^b^	KP-TD; p = 0.035
**Parousity**	83	False	-158.127	324.25	0.58	0.59	0.63	No difference between means (p>0.05)

### Biting cycles of *An*. *balabacensis*

**Fig 3:**
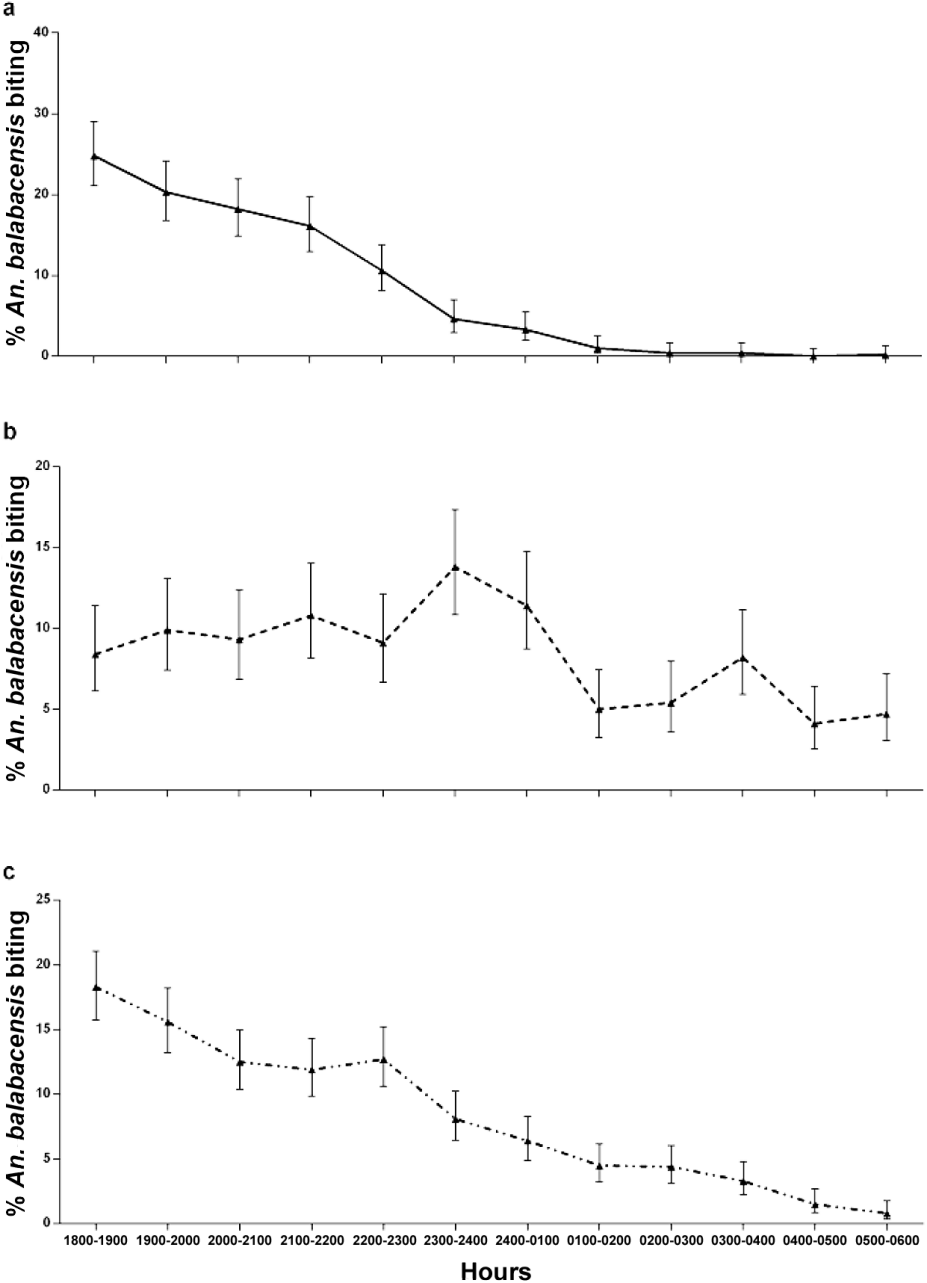
Percentage of An. balabacensis (out of the total collected) that were captured biting at each hour of the nightly sampling period (18:00–6:00hrs)in each study sites (pooled across all months of collection). a. Limbuak Laut, b. Timbang Dyang, c. Kg Paradason. Error bars are 95%CI.

As shown in Fig 3 *An*. *balabacensis* started to bite as early as 1800 hours and continued to bite throughout the night until early hours of the morning. The peak biting time occurred between 1800 to 2000hrs in both LL and KP (Fig 3A and 3C), accounting for 38% of the total night catch. In TD, biting rates were relatively similar between 1800-2400hrs, then began to fall with a second small peak in the early part of the morning (0300-0400hrs, Fig 3B).

### Transmission efficiency of *Anopheles balabacensis*

**Fig 4:**
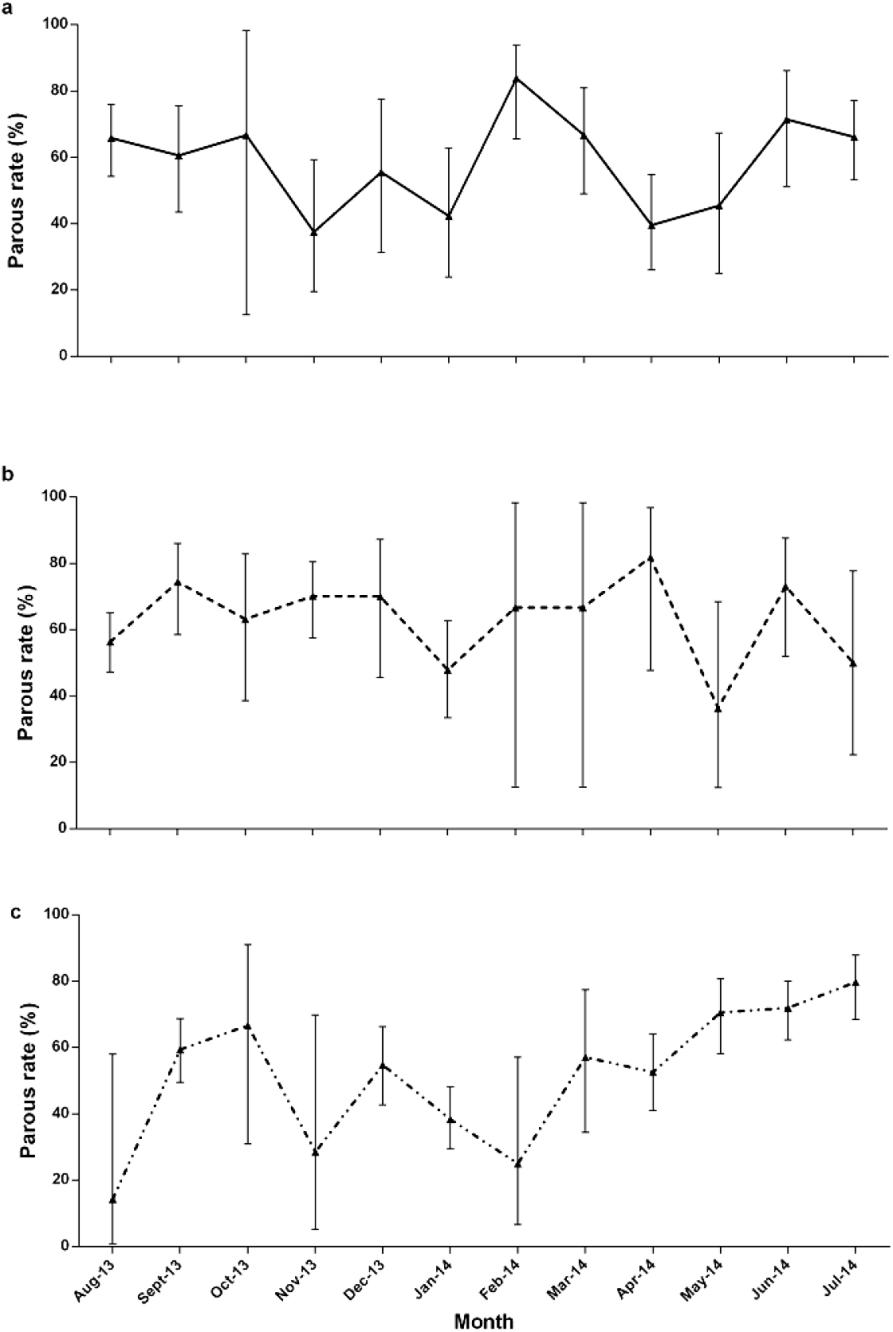
Parous rates (with 95% confidence interval) in three study sites. Alphabet denotes study sites: ^a^Limbuak Laut; ^b^Timbang Dayang; ^c^Kg Paradason.

The parous rate of *An*. *balabacensis* was more than 50% on most collections, in all sites (Fig 4). The mean parous rate varied between 58 to 65%, with little fluctuation (Fig 4, Table 3). Statistical analysis indicated no evidence of significant variation in parity rates between all 3 sites (p>0.05, Table 2). On the basis of the parous rate, a daily survival rate [38], life expectancy [39] and vectorial capacity values were calculated [40] for *An*. *balabacensis* at each site. Estimates of *An*. *balabacensis* survival and vectorial capacity were predicted to be higher in LL and TD compared to KP (Table 3). In LL and TD respectively, 24% and 22% of *An*. *balabacensis* would be expected to live the 10 days necessary for *P*. *knowlesi* to develop into transmission-stage sporozoites, contrasting with only 16% in KP. Those surviving the 10 days would have a further life expectancy of 7 and 6.7 days in LL and TD respectively, compared to 5.4 days in KP. Vectorial capacity was predicted to be highest in LL with an estimated value of 3.85.

**Table 3:** Annual infection rates, man-biting rate, entomological inoculation rate, parous rate, probability of daily survival, life expectancy and vectorial capacity of *An*. *balabacensis* in study sites.

	Banggi Island		Kudat
	Limbuak Laut	Timbang Dayang	Kg Paradason
Sporozoite rate (95%CI)	3.42 (1.91–5.93)	1.93 (0.85–4.12)	1.03 (0.45–2.20)
Oocysts rate (95% CI)	3.16 (1.72–5.60)	3.23 (1.76–5.74)	1.03 (0.45–2.21)
Man-biting rate (ma)	7.0	6.8	8.8
Entomological inoculation rate (EIR)	0.24	0.13	0.09
Parous rate (95% CI)	65.00 (59.94–69.75)	63.78 (58.63–68.65)	57.53 (53.68–61.29)
Probability of daily survival-p^1^	0.87	0.86	0.83
p^10^(%)	24	22	16
Life expectancy^2^	7.0	6.7	5.4
Vectorial capacity^3^	3.85	3.36	2.50

^1^The probability of daily survival (p) was taken as ^3^√P (P = percentage parous) (25).

^2^life expenctancy = p10/-log_e_ p (days) (26).

^3^Vectorial capacity (VC) = ma^2^p^n^/-log^e^p (27).

p^10^ Percentage of population expected to live long enough to become infective with an extrinsic cycle of 10 days based on *P*. *knowlesi* extrinsic incubation period

### Oocyst, sporozoite and entomological inoculation rates by months and sites

Forty five (3%) *An*. *balabacensis* out of the 1482 dissected were found to be positive for *Plasmodium* infection in terms of either sporozoites (14), oocysts (18) or both (13) by microscopy. Of these only 10 salivary glands and three midguts were positive for *P*. *knowlesi* by PCR. Besides *P*. *knowlesi* other simian malaria parasites were also present as shown in Table 4. This shows that in addition to *P*. *knowlesi*, *An*. *balabacensis* is also a vector to other simian *Plasmodium* species as well.

**Table 4:** Species of *Plasmodium* identified from mid gut and salivary glands of the *An*. *balabacnesis*.

Plasmodium species	Mosquito organs	
	Midgut	Salivary gland
Pk	0	1
Pcy	7	6
Pin	5	5
Pk+Pct+Pcy+Pin	0	1
Pk+Pcy+Pin	0	4
Pk+Pin	2	2
Pk+Pcy	1	2
Pcy+Pin	8	4
Not identified to date	8	2
**TOTAL**	**31**	**27**

Key: Pk = *P*. *knowlesi*; Pcy = *P*. *cynomolgi*; Pct = *P*. *coatneyi*; Pin = *P*. *inui*; 14 *An*. *balabacensis* has salivary glands positive; 18 *An*. *balabacensis* had midgut positive and 13 had both midgut and salivary gland positive. Thus total 58.

**Fig 5:**
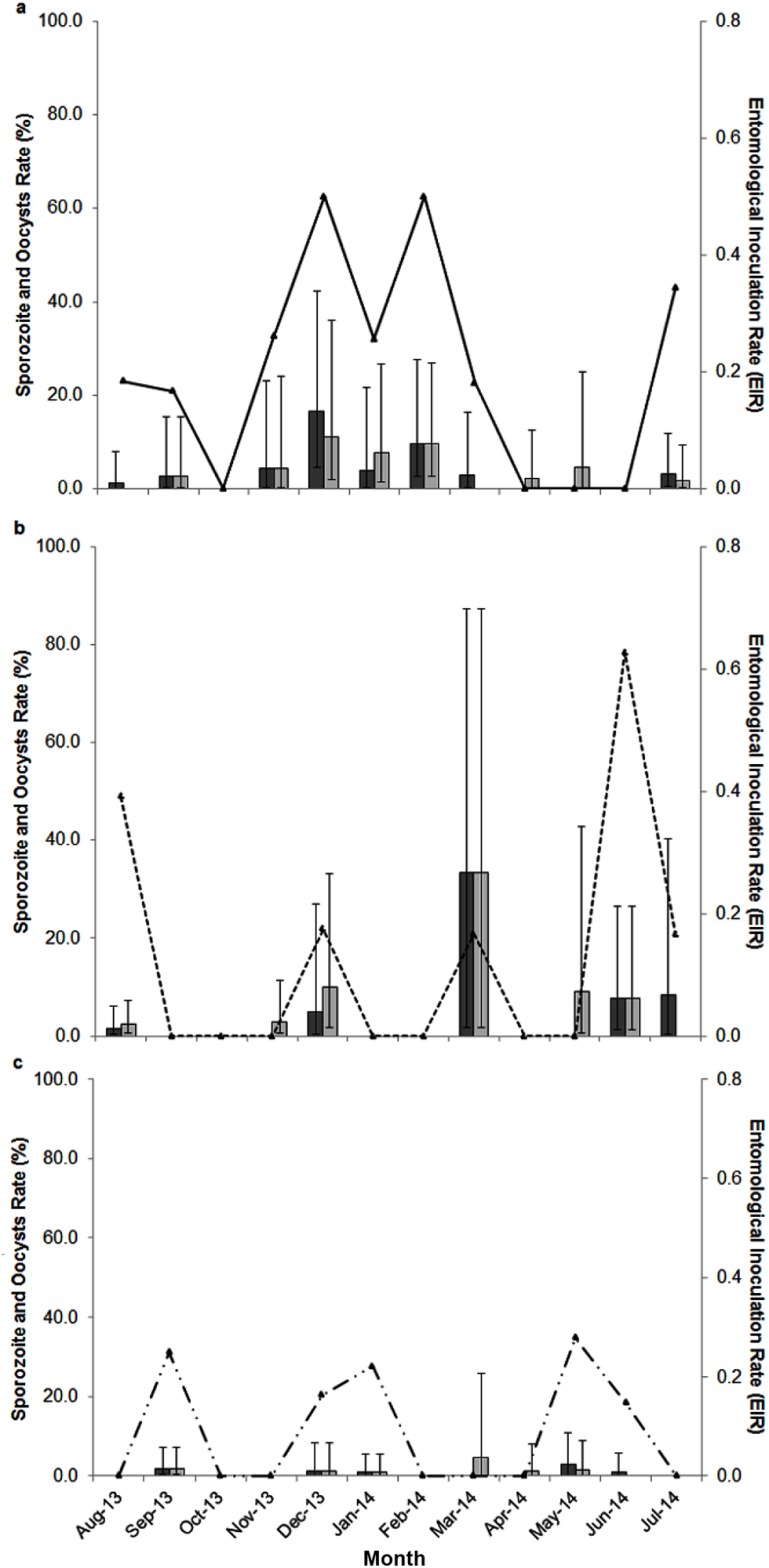
Infection rates and entomological inoculation rate (EIR) of *An*. *balabacensis* in the study sites. The solid blue line connects the points of EIR for *An*. *balabacensis*. Bars indicate the infection rates, which are sporozoite (dark grey with 95% confidence interval) and oocysts rate (light grey with 95% confidence interval). Alphabet denotes the study sites: ^a^ Limbuak Laut; ^b^Timbang Dayang; ^c^Kg Paradason.

Due complexity of infection the subsequent discussion refers to all Plasmodia. There was no consistent seasonal pattern of mosquito infection rates across sites (Fig 5). In LL sporozoite rates were highest from December to February (4–16.67%). In the TD, sporozoite rates were high in December (5.00%) and in June to July (7.69–12.50%). In March, only three mosquitoes at TD were dissected of which two were found to be positive; one for sporozoites and one for oocyst. Thus, sporozoite rates appear to be extremely high at this time, but it is likely an artifact of low sample size. In KP the highest sporozoite rate was obtained in May 2014 (2.86%). The highest entomological inoculation rate (EIR) was 0.6 in TD in June. Tukey post hoc tests performed on the results of statistical models of *An*. *balabancensis* infection rates indicated there was variation between sites. Specificially, sporozoites rates were lower in KP compared to LL (p = 0.04), and oocyst rates were lower in KP than in TD (p = 0.035) (Table 2). Sporozoite rates were estimated to be approximately 2 and 3 times higher in the LL and TD respectively than in KP (Table 2).

## Discussion

Our study provides the first evidence to confirm that *An*. *balabacensis* is the vector of the zoonotic malaria *P*. *knowlesi* within the substantial foci of human infection in Sabah. It was the predominant species found in all sites with mean biting rates ranging from 6.8 to 8.8. A substantial proportion of *An*. *balabacensis* (32.8%) were captured biting outdoors in the early part of the evening (1800–2000), a time when humans would not be expected to be using LLINs, which is the current front line malaria control strategy in Malaysia. In this study all collections were made outdoors, as previous studies have found that this is where the majority of *An*. *balabacensis* (~76%) host seek [28, 41]. However, we note that total amount of human exposure to infectious bites from *An*. *balabacensis* may be even higher than indicated here if the additional contribution of limited indoor exposure were to be incorporated. In comparing the density and bionomics of *An*. *balabancensis* populations between three sites, we found evidence of geographical variation in both their abundance and sporozoite infection rate. Vector abundance was highest in the village site, whereas sporozoite rates were higher in the forest and small farming site than in the village site. However, it is unknown whether these differences are truly the result of habitat-dependent transmission efficiencies, as only one site from each ecotype was sampled. However these findings reinforces the hypothesis that spatial heterogeneity in *P*. *knowlesi* exposure risk may be driven by variation in mosquito vector demography in addition to the presence of the reservoir macaque host.

Although it has been postulated that *P*. *knowlesi* was present in macaques before the arrival of humans in Southeast Asia [42], and a large number of *P*. *knowlesi* malaria cases has been reported from Sabah [20], the identity of the vector remained elusive. Whilst it has been demonstrated by Chin et al [43] that *An*. *balabacensis* can transmit *P*. *knowlesi* from monkey to man, man to monkey and man to man under experimental conditions, this study is the first to confirm that it acts as a vector under natural conditions. *Anopheles balabacensis* was also incriminated as the primary vector of human malaria in Sabah in the 1950s [44, 45]; a role that was further supported with extensive studies in the 1980s which confirmed its role as the main vector for human malaria infections [28, 46]. Given *An*. *balabacensis* is the likely vector of other primate malaria species in this area, it could also be the conduit for other zoonotic malaria spillovers to humans. This indicates that these *Plasmodia* species are not partitioned amongst different vector species, and emphasizes that *An*. *balabacensis* should be the primary target for all malaria control efforts in the area.

We observed a significant difference in the *Anopheles* species composition found here relative to previous studies in Sabah. Currently *An*. *balabacensis* and *An*. *donaldi* constituted > 95% and 1.3% of all *Anopheles* recorded on Bangii island respectively, while studies in this area in the 1980s estimated the relative proportion of these species to 13.6%, and 39% of *Anophele*s respectively [29]. In the central region of Sabah *An*. *donaldi* was incriminated as the dominant vector for human malaria parasites in studies carried out in 2001–2002 [41]. We did not document infection in *An*. *donaldi* within this study, but this may because too few were collected (n = 25) for reliable detection. Thus, we cannot dismiss the possibility that *An*. *donaldi* remains in other areas of Sabah where it is most abundant. The cause of this apparent shift in malaria vector species composition over the past 40 years in Banggi Island is uncertain although it coincides with a period of extensive deforestation in Sabah [33, 34]. One possibility is that this is just an artefact of sampling, as here we did not conduct sampling in the exact same locations as historical studies, but instead targeted sites of known human *P*. *knowlesi* infection. These sites may have inherently higher densities of *An*. *balabancensis* (thus triggering *P*. *knowlesi* infection) than other locations within the area. However, there is grounds to hypothesize this could be evidence of long-term shift in species composition in response to the rapid deforestation or prolonged use of interventions such as LLINs or IRS as has been documented elsewhere [47]. In previous work within the Kinabatangan area of Sabah, we have also documented a shift from a high proportion of *An*. *balabacensis* to dominance of *An*. *donaldi* within the same sites over the period 1980s to 2000 [29, 41]. Regardless of the explanation for the dominance of *An*. *balabacensis* within this study the relatively high survival and sporozoite rates in this vector coupled with the potentially increased contact of human-vector-macaques have likely made major contributions to the increase in *P*. *knowlesi* cases in the area.

Although Sabah has reported a large number of *P*. *knowlesi* cases in the past few years especially in Kudat district, it is hypothesized that people are only getting infected when they visit forested areas. Within our current study sites, the number of malaria cases occurring over the sampling period ranged from 1.9 to 2.5 cases per 100 people [48]. As positive *An*. *balabacensis* were present in most months of the year and most of the infective mosquitoes (40%) were captured biting in the early part of the evening between1800 to 2000, people could be exposed when they return from work in or around forested areas. Our preliminary studies now and previously have demonstrated that the *Anopheles* mosquitoes start biting only after 1800 hrs. The average biting rate reported for *An*. *balabacensis* here is much higher than in previous studies conducted in the 1980s (eg. 6.8 to 8.8/night compared to 0.75 to 4.44) [28]. These biting rates are also considerably higher than has been reported for *An*. *latens* (0.95 to 4.71 bites per night) in Sarawak [22]. The high density of *An*. *balabacensis* in this area combined with its relatively high sporozoite rates with all simian malaria (1.82%) and *P*. *knowlesi* in particular (0.67%) indicate it is most likely responsible for the majority of transmission in this area.

In this study all mosquitoes were collected using human bait, thus results are only directly informative for estimating potential human exposure and not transmission between macaques. Ideally parallel collections of mosquitoes attracted towards macaques would have been conducted but this was not possible due to logistical constraints and ethics regulations for working with macaques. Previous work [22, 23] showed that the *P*. *knowlesi* vectors in other areas namely *An*. *latens* and *An*. *cracens* were attracted to both humans and macaques. Furthermore in Palawan Island, Philippines, *An*. *balabacensis* was more attracted to a monkey baited trap than traps baited with water buffalo or humans and individuals host seeking on macaques had oocyst and sporozoites (although malaria species unconfirmed) [49]. Thus, although data for mosquitoes biting macaques are not available here, we could expect, that transmission between macaques to be at least as high or much greater than predicted for humans here.

To further resolve the transmission dynamics of *P*. *knowlesi* in primates, these studies should be expanded to incorporate assessment of the host preference and choice of *An*. *balabacensis* and other potential vectors most directly through analysis of the blood meals in randomly sampled resting females [50]. However, collection of recently blood fed mosquitoes resting outdoors has proved challenging. To overcome this limitation ongoing work is also investigating the use of new sampling methods to increase feasibility of such data collection in the future.

The high rate of parity, survival and sporozoite infections in this mosquito indicates that *An*. *balabcensis* is a highly competent vector. With a very high vectorial capacity and life expectancy, A*n*. *balabacensis* will continue to pose a risk of human infection. As Malaysia moves towards malaria elimination, breaking transmission under these conditions will be extremely challenging, further complicated by the presence of a sizeable macaque reservoir.

Current frontline malaria control measures in this area are insecticide treated bednets and indoor residual spraying but more innovative control methods that specifically target outdoor biting mosquitoes such as the use of repellents or attractive toxic sugar baits will be essential.
